# Health-related quality of life and survival of patients with hepatocellular carcinoma treated with transarterial chemoembolization and Yttrium-90

**DOI:** 10.1186/s43046-025-00267-1

**Published:** 2025-04-14

**Authors:** Kathryn Bress, Patrick Bou-Samra, Cramer J. Kallem, Allan Tsung, Ellie Gammer, David A. Geller, James W. Marsh, Jennifer L. Steel

**Affiliations:** 1 Department of Surgery, University of Pittsburgh Medical Center, Pittsburgh, PA USA; 2 Department of Psychiatry, University of Pittsburgh, Pittsburgh, PA USA; 3 Department of Psychology, University of Pittsburgh, Pittsburgh, PA USA

**Keywords:** Health-related quality of life, Survival analysis, Hepatocellular carcinoma, Transarterial chemoembolization, Radioembolization

## Abstract

**Background:**

Hepatocellular carcinoma (HCC) is the fifth most common cancer worldwide. Due to the advanced stage in which HCC presents, most patients are only eligible for transarterial chemoembolization (TACE) or radioembolization (Y^90^). The purpose of this study is to examine the differences in survival and health-related quality of life (HRQOL) in patients diagnosed with HCC and treated with TACE or Y^90^.

**Methods:**

Two hundred thirty-four patients with HCC were enrolled in studies examining HRQOL between 2003–2009. HRQOL was evaluated using the Functional Assessment of Cancer Therapy-Hepatobiliary (FACT-Hep). Between-group differences were examined using chi-square and ANOVA. Survival was assessed using Kaplan–Meier and Cox regression analyses.

**Results:**

Significant baseline differences between patients treated with TACE versus Y^90^ were found. Patients who received Y^90^ tended to be older (*p* < 0.001), female (*p* < 0.001), had fewer lesions (*p* = 0.03), had smaller tumors (*p* = 0.03), and were less likely to have vascular invasion (*p* = 0.04). After adjusting for demographic and disease-specific factors, no significant differences in HRQOL were observed at 3 months (*p* = 0.79) or 6 months (*p* = 0.75). Clinically meaningful differences were found, with the TACE group reporting greater physical, social, and emotional well-being at 3 and 6 months and greater overall HRQOL at 6 months. No significant differences in survival were found.

**Conclusions:**

Treatment with TACE and Y^90^ was similar with regard to survival. However, TACE showed statistically and clinically meaningful benefits in physical, social/family, and emotional well-being. Further research is warranted to identify profiles of patients who may demonstrate a preferential response to either TACE or Y^90^.

## Abstract (plain language summary)

Hepatocellular carcinoma (HCC) is the fifth most common type of cancer worldwide. For most patients with HCC, two procedures known as transarterial chemoembolization (TACE) and radioembolization (Y^90^), represent their most promising treatment options. The purpose of this study is to examine the differences in survival and health-related quality of life (HRQOL) in patients diagnosed with HCC and treated with TACE or Y^90^. In this sample, patients treated with TACE reported better HRQOL in the domains of physical, social, and emotional well-being at 3- and 6 months post-treatment and greater overall HRQOL at 6 months. There were no significant differences in patient survival based on the type of treatment they received. More research is needed to identify what treatment factors and patient characteristics might be associated with better HRQOL after being treated with TACE or Y^90^.

## Background

Worldwide, hepatocellular carcinoma (HCC) is the fifth most common cancer, with 560,000 new cases annually [1, 2]. Resection and transplantation are the only curative options for HCC; however, the majority of patients (> 80%) cannot undergo these surgical options secondary to the risk [3, 4]. To potentially extend survival and improve quality of life, regional treatment methods are recommended. The two primary regional treatments patients receive include transarterial chemoembolization (TACE) and radioembolization with yttrium-90 (Y^90^) [5, 6]. Both of these options involve hepatic arterial infusion of a therapeutic substance with the use of percutaneously placed catheters [7]. No randomized controlled trial has been performed testing the superiority of these treatment options.

Chemoembolization delivers a dose of a therapeutic drug or combination of drugs, most commonly Adriamycin, Mitomycin C, cisplatin, or a combination of these drugs [8, 9]. Trials of chemoembolization have had varied results, mostly reporting a marginal increase in survival, accompanied by toxicity. Side effects of TACE can include abdominal pain, fever, nausea, vomiting, cholecystitis, hepatic abscess, bleeding, hepatic decompensation, or fulminant liver failure. The more severe of these side effects are very low risk, affecting less than 2% of patients receiving treatment [10–13]. In a study with patients diagnosed with HCC and receiving TACE, 44% of patients experienced toxicity, with 29% of patients experiencing grade 3 or higher toxicity [14].

Radioembolization, a more recent alternative, delivers glass microspheres which have been infused with a beta-emitting radioactive compound, most commonly Y^90^. Radiotherapy in a high enough dose is effective in treating HCC but historically has been limited by the inability of the healthy liver tissue to survive the treatment without complication [15]. It exploits the pathophysiology of HCC, which derives almost all of its blood supply from hepatic arteries as opposed to the native liver parenchyma which depends predominantly on portal venous supply [1]. This difference in perfusion pattern allowed tumoricidal doses of radiation to be locally administered and targeted to the tumor cells with relative sparing of a healthy liver.

Though the concept of directed radiotherapy is not new, Y^90^ treatment has not always been widely accepted. Early trials with Y^90^ showed severe toxicity and only marginal improvements in survival were observed [15–17]. However, in some cases, TARE has been effective in sufficiently reducing tumor volume to the point that surgical resection becomes a feasible option [18]. Patients with larger tumors are better treated with radiation because of the high energy and depth of permeation accompanying the treatment [19, 20]. In the case of Y^90^, the maximum tissue permeation is estimated to be 11 mm [21]. Patients with existing liver damage may also benefit from radioembolization, as it may cause less damage to hepatocytes [22].

Kooby, et al. examined the difference in treatment effectiveness and toxicity in 71 patients treated with either TACE or Y^90^ radioembolization [14]. This study found no significant difference in survival between treatment groups. However, a significant difference in hospital stay length and severity of toxicity was observed with radioembolization resulting in lower toxicity and shorter hospital stay. Definitive conclusions regarding the benefit of Y^90^ have not been possible secondary to the small sample sizes in many studies and lack of randomized controlled trials.

Salem and colleagues examined HRQOL in 56 HCC patients following either Y^90^ radioembolization or TACE and found no statistically significant differences in health-related quality of life (HRQOL) or survival between patient groups [23]. To our knowledge, no other studies have examined differences in HRQOL between radioembolization versus chemoembolization despite these treatments being employed extensively worldwide. This prospective cohort study will examine HRQOL and survival in a larger sample of HCC patients prior to and following treatments of chemoembolization and radioembolization with Y^90^.

## Methods

### Design

The study was prospective in design and a secondary data analysis. Patients were enrolled in these studies between September 2002 and August 2009 [24, 25].

### Participants

The current study utilized data from two existing studies. All patients were enrolled in an outpatient oncology clinic at a medical center. Inclusion criteria for the patients were (1) biopsy, radiological and/or biological evidence of hepatocellular carcinoma, (2) age 21 years or older, (3) fluency in English, and (4) patients met the medical criteria and were treated with TACE or Y^90^ for treatment. Exclusion criteria included: (1) age under 21 years, (2) current suicidal or homicidal ideation, or current psychosis or thought disorder, and (3) resection or transplant candidate. In the two original studies, 424 patients were approached and 345 consented to participate. Of those, 111 patients were not treated with TACE or Y^90^ and were excluded from this study, resulting in a final sample of 234 (144 treated with TACE and 90 with Y^90^).

### Sociodemographic and disease-specific factors

A 25-item questionnaire was administered to collect patients’ sociodemographic information. The disease-specific and treatment-related data was collected through the patients’ electronic medical records. Survival was calculated from the date of evaluation (diagnosis) to death. Death was collected from the Social Security Death Index.

### Instruments

The Functional Assessment of Cancer Therapy (FACT)-Hepatobiliary was used to assess the quality of life of the cancer patient [26, 27]. The FACT-Hep includes both the FACT-General (FACT-G) and a module specific to hepatobiliary disease. The FACT-G is a 27-item instrument with four subscales for physical (PWB), social and family (SFWB), emotional (EWB), and functional well-being (FWB). The hepatobiliary module (FACT-Hep) includes 18 items that pertain to symptoms of the disease as well as side effects of treatment. The FACT is one of the most widely utilized quality-of-life questionnaires in clinical trials for new cancer treatments. The FACT-Hep have been demonstrated to be valid and reliable instruments as they have been shown to be accurate and precise measures of health-related quality of life (HRQOL), especially with hepatopancreatobiliary cancers (HPB) [26, 27]. Clinically meaningful differences have also been established for the FACT-Hep [28].

### Procedure

The study was approved by the University of Pittsburgh Institutional Review Board (IRB). Patients were evaluated and treated at a large tertiary cancer center and were referred by their attending physician. The patient provided written informed consent prior to administration of the questionnaires. Questionnaires were sent to the patient with a self-addressed stamped envelope.

### Treatment

TACE is a combination of embolization and local chemotherapy. The embolotherapy interrupts the arterial blood flow to the liver parenchyma impeding the washout of the injected chemotherapy [29]. The selective embolization induces local tumor tissue necrosis [29]. TACE treatment was performed using standard chemoinfusion protocol used at our institution using either Cisplatin followed by embolization or using a combination of 100–300- and 300–500-micron particles of Embosphere microspheres (Merit Medical, South Jordan, UT, USA). Therapy was repeated in 6–8 weeks as determined by follow-up cross-sectional imaging.

### Yttrium 90

Standard procedures for Y^90^ radioembolization administration were performed. Pre-treatment dosimetry was performed on every patient by interventional radiology and nuclear medicine. Patients were treated with Y^90^-impregnated glass microspheres (half-life of 2.67 days, mean diameter 20–30 µm), delivered to patients via transfemoral catheterization of the hepatic artery with a catheter of internal diameter ≥ 0.5 mm (0.020 inches) [30, 31]. A constant syringe pressure using a flow rate of no less than 20 cc per minute is needed and should be continued until optimal delivery is achieved. Following the administration into the hepatic artery, these microspheres remain in the terminal arterioles of the tumor vasculature and gradually decay delivering the required radiation locally. Their high specificity to the tumor cells entails less required dosages to be administered with equal efficacy. Also, their targeted distribution provides more thorough coverage to the tumor while sparing normal tissues. For the purposes of this study, the post-treatment distribution of TARE was not evaluated.

### Statistical analyses

Data were imported from a website that was utilized for data collection and verified in SPSS.v21 for analyses. Missing data were checked to determine if random. All data were found to be missing at random and, for cases with less than 50% missing, the values were replaced using the mean of the observed value for that variable. Descriptive statistics were employed to examine the distribution of data and provide the characteristics of the sample. Multivariate analysis of variance (MANOVA) was performed to test differences between treatment groups and Kaplan–Meier and Cox regression survival analyses were performed to test the association between sociodemographic, disease-specific, and treatment factors.

## Results

### Clinical characteristics and baseline differences

A total of 234 patients diagnosed with HCC were included in the study. The mean age of the sample was 65.9 years, and the majority of the patients were male (76%). The mean tumor size was 6.9 cm and the mean number of lesions was 3.3. Forty-five percent of the patients had vascular invasion. The patients who were treated with TACE versus Y^90^ were significantly different at the time of the first treatment with regard to age [*F*(1,199) = 12.8, *p* < 0.001], gender [chi-square = 12.6, *p* < 0.001], number of lesions [*F*(1,217) = 4.6, *p* = 0.03], tumor size [*F*(1,214) = 5.1, *p* = 0.03], and vascular invasion [chi-square = 4.1, *p* = 0.04]. Patients who received Y^90^ were older (69.7 versus 63.7 years), more likely to be female (37% versus 17%), had fewer number of lesions (2.9 versus 3.5), more likely to have smaller tumors (6.1 versus 7.6 cm), and less likely to have vascular invasion (25% versus 55%). No difference in the presence of cirrhosis was observed between the two treatment groups. See Table 1 for sociodemographic and disease-specific characteristics.

**Table 1:** Sociodemographic and disease-specific characteristics

Sociodemographic and disease-specific characteristics (*n* = 234)				
	Entire sample	TACE	Y^90^	*p*-value
Age (mean, SD)	65.9	63.7 (12.4)	69.7 (10.5)	< 0.001
Gender (*n*; % male)	178 (76)	119 (83)	55 (63)	< 0.001
Cirrhosis (*n*, %)	201 (86)	98 (86)	61 (87)	0.935
Number of lesions (mean; SD)	3.3 (2.1)	3.5 (2.1)	2.9 (2.0)	0.03
Tumor size (mean, SD)	6.9 (4.8)	7.6 (5.0)	6.1 (4.4)	0.03
Vascular invasion (*n*, %)	105 (45)	58 (55)	26 (25)	0.043

### Health-related quality of life

Baseline differences in HRQOL were analyzed. No significant differences were observed between patients who received TACE versus those who received Y^90^ on the physical [*F*(6,186) = 0.41, *p* = 0.87], social and family [*F*(6,189) = 0.43, *p* = 0.86], emotional [*F*(6,200) = 0.33, *p* = 0.92, functional well-being [*F*(6,183) = 0.38, *p* = 0.89], the symptoms and side effects [*F*(6,180) = 0.83, *p* = 0.55] or overall HRQOL [*F*(6,170) = 0.63, *p* = 0.71].

### Treatment group differences in HRQOL at 3- and 6 months

**Fig. 1:**
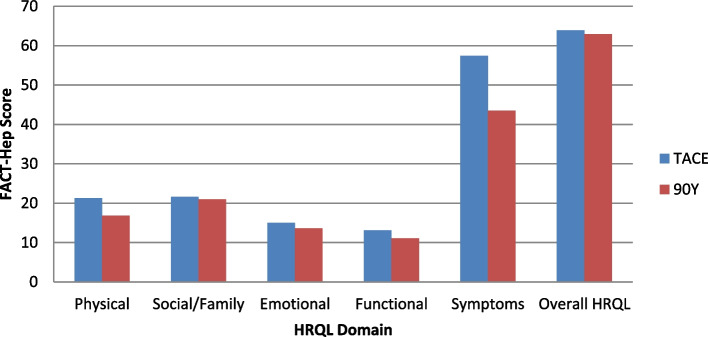
Between treatment group differences in health-related quality of life at 3 months follow-up

Multivariate analyses of variance (MANOVA) were performed adjusting for factors that were significant at baseline (e.g., age, gender, number of lesions, tumor size, and vascular invasion). We found no significant differences between the treatment groups at 3 months on the five domains of the FACT-Hepatobiliary [Wilk’s Lambda *F*(5, 75) = 0.47, *p* = 0.79] or 6 months [Wilk’s Lambda *F*(5, 52) = 0.51, *p* = 0.75] (Fig. 1).

**Fig. 2:**
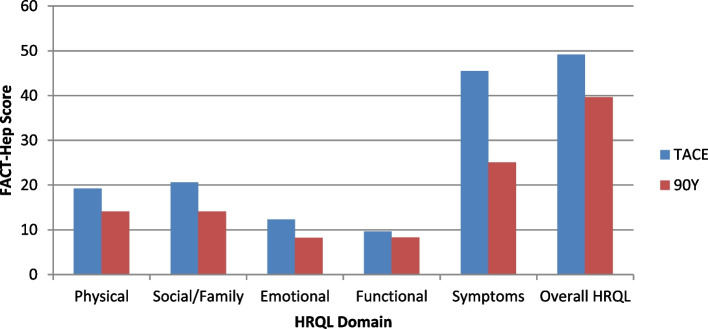
Between treatment group differences on health-related quality of life at 6 months

Although there were no statistically significant differences at 3 months, clinically meaningful differences were observed at 6 months on the physical well-being domain with patients treated with TACE having better mean PWB scale score (19.8 versus 13.4). Clinically meaningful differences were also observed in which patients treated with TACE had better social and family well-being (20.1.9 versus 13.6) and emotional well-being (12.7 versus 9.8). Clinically meaningful differences were not observed for the functional (9.9 versus 8.6) or symptoms and side effects domains of the FACT Hepatobiliary (45.9 versus 241.3) or overall HRQOL (49.2 versus 40.0). See Fig. 2.

### Treatment of HCC and survival

Univariate Kaplan–Meier and Cox survival analyses were performed for each of the sociodemographic and disease-specific factors and HRQOL. No significant differences were found in survival with regard to treatment [log rank chi-square = 0.20, *p* = 0.65], gender [log rank chi-square = 0.10, *p* = 0.75]; vascular invasion [log rank chi-square = 0.85, *p* = 0.36]; age [chi-square = 0.015, *p* = 0.90]; and lesion number [chi-square = 0.031, *p* = 0.86]. Significant differences were found between those patients who had cirrhosis and those who did not with regard to survival [log rank chi-square = 4.3, *p* = 0.04] and tumor size [chi-square = 4.5, *p* = 0.04]. A Cox regression analysis was performed adjusting for significant predictors of survival including cirrhosis and tumor size. The overall model and the predictors included in the model were not found to significantly predict survival [chi-square = 6.4, *p* = 0.09]. See Table 2.

**Table 2:** Cox regression analyses of predictors of survival

	Beta	SE	*p*-value	HR	95% CI	
					Lower	Upper
Cirrhosis	1.141	.807	.157	3.129	.644	15.210
Tumor size	− .087	.061	.153	.916	.813	1.033
Treatment	− .200	.438	.647	.818	.347	1.930

## Discussion

Existing therapeutic options such as chemoembolization and radioembolization have been shown to significantly increase survival [22, 32, 33]. Each option potentially has an optimal set of conditions that would give rise to its preferential administration; however, current indications and contraindications for TACE and Y^90^ are largely similar; therefore, the administration is often based on clinical decision-making making which varies between physicians and centers [32]. Comparative studies of TACE and Y^90^embolization can support more uniform treatment criteria. Increasing focus on HRQOL may be beneficial, given the established lack of differences in survival. A paucity of comparative studies between these treatments exists. It is worth noting that there are important differences between these approaches. The effectiveness of TARE, both in terms of survival and quality of life, depends heavily on the accuracy of pre-TARE planning [34]. Future studies should also consider the effects that personalized dosimetry planning may have on health-related quality of life.

Two studies have examined tumor response, survival, and toxicity in HCC patients treated with TACE or Y^90^ [14, 33]. Both of these studies showed no significant difference in survival between treatment arms. Lance and colleagues showed significantly higher complication rates for patients treated with TACE while Kooby and colleagues reported no significant difference in toxicity between the two treatments [14, 33]. Salem and colleagues examined HRQOL in 56 HCC patients following either Y^90^ radioembolization or TACE and found no statistically significant differences in HRQOL or survival between patient groups [23].

The strength of the present study was the larger sample size and inclusion of a valid and reliable measure of HRQOL. There was no significant difference in survival between the two treatment groups using univariate or multivariate analyses; however, this was not a randomized controlled trial so definitive conclusions regarding the survival benefits of these treatments cannot be made at this time. These findings were consistent with prior clinical trials comparing TACE and Y^90^ [33, 35]. Early trials also reported little difference with regard to side effects; patients treated with Y^90^ most commonly developed transient fatigue, diarrhea, anorexia, nausea, vomiting, fever, and abdominal pain more often than TACE patients [33, 35]. Post-embolization syndrome, associated with TACE, includes a similar symptom cluster as Y^90^ side effects, but the severity of symptoms is greater and more debilitating, for some patients requiring extended hospitalization [36].

In this study, HRQOL was higher in all of the domains, including overall HRQOL, for patients administered TACE despite the lack of differences in HRQL prior to treatment. Clinically meaningful differences were only observed in the physical, social-family, and emotional well-being domains. At 6 months a clinically meaningful difference was also observed in overall quality of life for patients treated with TACE when compared to Y^90^. A clinically meaningful difference is “the smallest difference in score in the domain of interest that patients perceive as important, either beneficial or harmful, and which would lead a clinician to consider a change in the patient’s management [28].”

The physical well-being domain of the FACT-G includes symptoms such as nausea and pain, malaise and nausea, and spending more time in bed. The social and emotional well-being subscales reflect the patients’ feelings of support from family and friends and symptoms of anxiety and depression, respectively. These were all greater in those receiving TACE when compared to those patients receiving Y^90^. Given the lack of significant differences in symptoms and side effects between groups, the difference in overall physical well-being is most likely not secondary to a difference in side effects from the treatments but may be related to changes in immunologic function post-treatment, which would affect the body systemically [37, 38]. Other studies involving biomarkers of inflammation following embolization have demonstrated immunosuppression in HCC patients following treatment with TACE and RFA [38, 39]. Research on similar immune effects in patients treated with Y^90^ is limited. Given the difference in physical well-being, it is plausible that immunosuppression may be more severe following Y^90^ treatment. A comparative study of immune function in patients following each embolization therapy would help in identifying possible mechanisms for the observed difference in physical well-being between treatment groups. The timing of administration of the HRQOL instruments is also approximately 7–8 weeks after treatment. Therefore, the immediate side effects of treatment are not being captured. Measurement of immediate post-treatment changes in HRQOL may facilitate the understanding of potential differences in treatments.

Administration of Y^90^ requires additional procedures (e.g., planning angiogram) and this may have decreased the social-family well-being of the patients in the Y^90^ treatment arm. The cost-effectiveness of the treatments has not been examined and this may significantly add to the understanding of the benefits of these treatments.

Further study of HRQOL in the context of disease etiology and progression would also be beneficial. Different risk factors may have associated differences in treatment response and symptoms and side effects [40]. For example, physical well-being may be different for patients with alcohol-related HCC versus HCC with hepatitis C as the major contributing factor in the development of cirrhosis and HCC. Furthermore, etiology affects patient characteristics, which would also be expected to lead to differences in social, functional, and emotional well-being [40]. For example, those with alcohol-related HCC may have a smaller social network and/or lower levels of emotional well-being when compared to patients with non-alcoholic steatohepatitis.

Further research is warranted evaluating differences in survival and HRQOL in patients receiving systemic treatment such as sorafenib, sequential treatment with both radio- and chemo-embolization [41–43]. Cisplatin has been shown to have a higher response rate in combination with either doxorubicin, mitoxantrone, or 5-FU [41–43]. Studies have not examined HRQOL with these combined treatments as it would be expected that they may have increased levels of toxicity. Due to the expected increased prevalence of HCC, further research is needed to understand the potential factors that contribute to increased response to treatment and quality of life.

## Conclusions

In this prospective study, differences in HRQOL and survival were examined in a cohort of patients who were diagnosed with HCC and treated with TACE or Y^90^. There were no significant differences with regard to survival. However, TACE showed clinically meaningful benefits in physical, social/family, and emotional well-being. This may offer preliminary evidence that, on average, HCC patients who are treated with TACE tend to rate their HRQOL higher than patients treated Y^90^. However, because this was not a randomized controlled trial, definitive conclusions regarding the comparative efficacy of these treatments cannot be made at this time. Further research is also warranted to identify profiles of patients who may demonstrate a preferential response to either TACE or Y^90^.

## Data Availability

The data that support the findings of this study are available from the corresponding author upon reasonable request. Data may be made available to qualified investigators with reasonable aims and hypotheses that were not tested as part of this study.

## Abbreviations

HRQOL: Health-related quality of life
HCC: Hepatocellular carcinoma
TACE: Transarterial chemoembolization
Y^90^: Yttrium-90/radioembolization
FACT-Hep: Functional Assessment of Cancer Therapy-Hepatobiliary
FACT-G: Functional Assessment of Cancer Therapy-General
PWB: Physical well-being
SFWB: Social and family well-being
EWB: Emotional well-being
FWB: Functional well-being
IRB: Institutional Review Board
NASH: Non-alcohol steatohepatitis
